# A standardized single-tube 17-color spectral flow cytometry workflow for integrated immunophenotyping of human PBMCs and mixed co-culture systems

**DOI:** 10.3389/fimmu.2026.1799028

**Published:** 2026-05-08

**Authors:** Klaudia Kiel, Marta Małuszek, Katarzyna Piwocka, Jakub Godlewski, Agnieszka Bronisz

**Affiliations:** 1 Tumor Microenvironment Laboratory, Mossakowski Medical Research Institute, Polish Academy of Sciences, Warsaw, Poland; 2 Translational Medicine Doctoral School, Centre of Postgraduate Medical Education, Warsaw, Poland; 3 Laboratory of Cytometry, Nencki Institute of Experimental Biology, Polish Academy of Sciences, Warsaw, Poland; 4 Department of NeuroOncology, Mossakowski Medical Research Institute, Polish Academy of Sciences, Warsaw, Poland

**Keywords:** activation markers, cytek aurora, full spectrum, immune-tumor organoids, PBMCs, single tube assay, spectral flow cytometry, spectral unmixing

## Abstract

We present a standardized single-tube 17-color spectral flow cytometry workflow for integrated immunophenotyping of human peripheral blood mononuclear cells (PBMCs). This Methods Article describes a quality-controlled protocol that combines lineage identification with activation, regulatory, and checkpoint-associated markers within a reproducible quality-control framework. Panel optimization incorporated spectral similarity index (SI) and spillover spreading matrix (SSM) metrics, antigen-density-guided fluorochrome assignment, systematic antibody titration, fluorescence-minus-one (FMO)-anchored gating, autofluorescence extraction, and longitudinal internal controls. The workflow supports robust immunophenotyping of PBMCs across experiments when appropriate viability gating, autofluorescence correction, and quality-control checkpoints are applied, and enables CD45-based compartment discrimination in mixed immune–tumor co-culture systems. Single-tube multiparameter staining facilitates unsupervised cluster analysis and supports identification of expected and potentially underappreciated immune subsets. The manuscript emphasizes methodological transparency, reproducibility strategies, and principles supporting panel transferability rather than hypothesis-driven biological discovery. This work provides a practical and transferable framework for high-dimensional immune monitoring in translational and preclinical settings.

## 1 Introduction

High-dimensional immunophenotyping of peripheral blood mononuclear cells (PBMCs) is central to translational immunology, vaccine evaluation, immune monitoring, and preclinical modeling. In both research and clinical contexts, robust and reproducible immune-profiling workflows are required to ensure comparability across experiments, operators, and acquisition days. While spectral flow cytometry expands multiplexing capacity by leveraging full emission spectra, increased panel complexity introduces analytical challenges, including spectral similarity, spreading error, fluorochrome instability, and gating variability. Despite growing adoption, standardized optimization and validation frameworks that integrate spectral metrics, antibody titration logic, and longitudinal controls remain underreported.

This manuscript was developed as a Methods Article/Protocol resource. Its purpose is to provide a benchmarked, quality-controlled, and reproducible single-tube workflow for integrated PBMC immunophenotyping, together with transparent documentation of optimization decisions and quality-control strategies, rather than to report hypothesis-driven biological discovery.

In routine clinical immunophenotyping, flow cytometry is most often implemented as low- to mid-parameter, indication-driven panels, frequently across multiple tubes, optimized for predefined diagnostic or monitoring questions (e.g., primary/secondary immunodeficiencies, hematologic malignancy workups, post-transplant immune monitoring, autoimmune/inflammatory disease profiling, and therapy monitoring). These clinical workflows emphasize assay validation, instrument standardization, and inter-operator reproducibility under quality-system constraints, as reflected in international guidance and laboratory standards for clinical flow cytometry (1). In onco-hematology, widely adopted frameworks such as EuroFlow exemplify standardized, algorithmic, multi-tube strategies that integrate screening and classification steps to maximize diagnostic robustness (2). Against this background, the workflow presented here is not intended to replace clinical diagnostic panels but to provide a compact, single-tube, quality-controlled framework that integrates lineage definition with interpretable functional state readouts (activation, regulation, checkpoint-associated markers), supporting reproducible immune monitoring in translational and preclinical settings, including mixed co-culture systems. The used strategy allows also for unsupervised clustering and advanced comparative cluster analysis. This is crucial for the above mentioned study as well as advanced diagnostics.

High-dimensional panels for PBMC immunophenotyping have been widely reported and serve as important reference frameworks for multi-lineage gating and immune monitoring. For example, several widely used panels provide a broad backbone for concurrent identification of major lymphoid and myeloid subsets (3). In contrast, later designs extend peripheral blood profiling to substantially higher marker counts for deeper phenotyping when sample volume is limited. Other panels have been built around specific functional themes, including inhibitory-receptor–focused designs (4), while ultra–high-parameter spectral implementations (5) demonstrate expanded coverage in whole blood. Collectively, these efforts emphasize either maximal dimensionality or a narrow functional focus, often at the cost of increased implementation and standardization complexity (5–7). In contrast, the present workflow prioritizes practical implementability as a single-tube, 17-marker spectral panel that retains a compact multi-lineage backbone while integrating interpretable state readouts (activation, regulation, and checkpoint-associated markers), and embeds an explicit quality-control strategy to support reproducible PBMC immunophenotyping across experiments.

Immune monitoring requires identifying major immune cell subsets and analyzing their dynamic functional states, especially activation, regulation, and checkpoint-associated phenotypes, which are crucial for understanding immune competence and guiding treatments (8). Existing multicolor panels often depend on multi-tube staining or are limited to specific compartments (e.g., T cells only), reducing their practicality for comprehensive, scalable immune profiling (9, 10). Conventional compensation-based flow cytometry approaches are prone to spreading error, especially when detecting weakly expressed markers such as TIM-3, PD-1, or FoxP3 (11, 12). In heterogeneous samples, cell–type–specific autofluorescence can further confound low-intensity signals, motivating workflows that explicitly address autofluorescence and spreading error during panel design and analysis. Spectral cytometry can address these issues by using full emission spectra, enabling better marker resolution, more flexible panel design, autofluorescence extraction, and more effective unmixing of overlapping fluorochromes (13).

To address the need for a reliable, unified workflow, a 17-color panel was designed that integrates a compact multi-lineage backbone with functional state markers in a single tube. These encompass key lineage markers (CD3, CD4, CD8, CD14, CD16, CD56, CD11c, CLEC4C, HLA-DR) and functional readouts for activation (CD25, CD69, CD83), checkpoint-associated markers (PD-1, TIM-3), and immune regulation (FoxP3). Such a design enables the single-tube classification of diverse subsets, including early- and late-activated CD4^+^ and CD8^+^ T cells, Tregs, activated and checkpoint-associated NK and NKT cells phenotypes, classical and non-classical monocytes, and plasmacytoid and classical DCs.

The panel was developed through iterative optimization to balance antigen density with spectral separation and minimize spreading error, especially for low-abundance or critical targets (14). To enhance reproducibility and reduce background artifacts, the workflow incorporates reagent and buffer choices intended to reduce non-specific binding (including on CD16) and to limit fluorochrome-dependent spreading artifacts, alongside systematic titration and FMO-anchored gating. The staining protocol also includes a viability dye and a fixation/permeabilization step to detect surface and intracellular markers simultaneously. A same-donor PBMC internal control is incorporated as a longitudinal reference to monitor technical stability across acquisition days. This anchor reference sample minimizes the batch-effect in the longitudinal studies.

In addition to PBMC-only assays, the workflow supports mixed co-cultures containing CD45^-^ non-hematopoietic cells (e.g., tumor or stromal lines). CD45-based compartment discrimination enables simultaneous immune phenotyping and consistent exclusion/monitoring of the CD45^-^ compartment within the same tube, without changes to the antibody cocktail or workflow. Where applicable, a matched CD45^-^ control sample can be used to anchor gates for the CD45^-^ compartment.

This article details the complete protocol, including antibody setup, titration strategy, gating hierarchy, and quality control measures. It highlights key applications of the panel, such as post-stimulation assays, checkpoint-associated marker profiling, vaccine response evaluation, and comparative analyses across donors or treatments. In this study, we tested the utility of the workflow using PBMC-GSC (glioblastoma stem-like cells) co-culture organoids as a representative multicellular model system. This workflow provides a practical blueprint for standardized, high-dimensional immune monitoring in translational and preclinical settings, including PBMC-only assays and CD45-partitioned mixed co-culture systems.

The novelty of this work lies not in the identification of new immune subsets, but in the systematic integration of: (i) spectral similarity index (SI) and spillover spreading matrix (SSM) metrics for fluorochrome assignment, (ii) antigen-density–guided panel design, (iii) antibody titration performed under final workflow conditions, (iv) fluorescence-minus-one (FMO)–anchored gating for dim markers, and (v) longitudinal internal PBMC controls to support inter-run comparability. Together, these elements form a reproducible and transferable workflow blueprint for high-dimensional immune monitoring in translational and preclinical settings using full spectrum cytometry.

## 2 Material and equipment

### 2.1 Biological material

#### 2.1.1 Source of cells

PBMCs were isolated from buffy coats obtained from healthy donors (Regional Blood Center, Warsaw) in accordance with institutional and national regulations, under protocols approved by the Ethics Committee of the Centre of Postgraduate Medical Education (WAW/111/2021). Freshly isolated PBMCs were used for panel optimization and validation. In addition, a cryopreserved, single-donor PBMC aliquot was included in each run as a longitudinal internal control.

GSCs were obtained from surgical resections at Brigham and Women’s Hospital under IRB-approved protocols (2013P001236). Informed consent was obtained from all patients before tissue collection. Cryopreserved GSCs were used.

#### 2.1.2 Cell culture

PBMCs were isolated from buffy coats by density gradient centrifugation using Lymphoprep™ according to the manufacturer’s instructions. After isolation, cells were cultured in RPMI-1640 medium supplemented with 10% fetal bovine serum (FBS) and 1% penicillin-streptomycin and incubated at 37°C in a humidified incubator with 5% CO_2_. Cell viability was assessed by trypan blue exclusion or Muse Cell Analyzer before experimental use.

GSCs were cultured under serum-free conditions in Neurobasal medium supplemented with B-27 without Vitamin A, GlutaMAX, recombinant human basic fibroblast growth factor (bFGF), and recombinant human epidermal growth factor (EGF). Cultures were maintained at 37°C in a humidified atmosphere with 5% CO_2_, using ultra-low attachment plates to promote neurosphere formation. Cell viability was assessed by trypan blue exclusion or Muse Cell Analyzer before experimental use.

#### 2.1.3 Cell cryopreservation and thawing

PBMC cryopreservation. For cryopreservation, cells were pelleted and resuspended at 50 × 10^6^ cells/mL in freezing medium consisting of 90% fetal bovine serum (FBS) and 10% dimethyl sulfoxide (DMSO), with a maximum of 75 × 10^6^ cells per cryovial. Cryovials were cooled at approximately −1°C/min using a controlled-rate freezing container (e.g., isopropanol-based) to −80°C, and subsequently transferred to liquid nitrogen for long-term storage.

PBMC thawing and preparation for staining. Cryovials were rapidly thawed in a 37°C water bath until a small ice crystal remained. Cell suspensions were immediately transferred to pre-warmed complete RPMI-1640 medium by slow, dropwise dilution to minimize osmotic shock, followed by centrifugation to remove DMSO. Cells were washed once more in complete medium and then resuspended in EasySep™ buffer. Cell viability and cell counts were assessed by trypan blue exclusion or Muse Cell Analyzer before staining. After DMSO removal and recovery, cryopreserved PBMC aliquots were thawed and stained using the same workflow as freshly isolated PBMCs.

GSC cryopreservation and thawing. Patient-derived GSCs were cryopreserved in Bambanker freezing medium according to the manufacturer’s recommendations and stored in liquid nitrogen. For recovery, cryovials were rapidly thawed at 37°C, and the cell suspension was diluted into pre-warmed culture medium, followed by centrifugation at 250 × g for 5min to remove freezing medium. The supernatant was discarded, and cells were resuspended in pre-warmed Neurobasal-based complete medium and returned to serum-free neurosphere culture conditions before use in co-culture experiments.

#### 2.1.4 Co-culture setup

PBMCs and GSCs, prepared according to the procedures outlined in Section 3.1.1, were combined at a 10:1 PBMC: GSC ratio. Co-cultures of organoids were maintained for 72h in RPMI-1640 medium supplemented with 10% FBS and 1% penicillin–streptomycin at 37°C in a humidified 5% CO_2_ incubator. Following incubation, co-culture organoids were collected, enzymatically dissociated with Accutase, washed, and processed for staining according to the unified flow cytometry protocol.

Co-culture conditions were selected as a short-term proof-of-compatibility for mixed CD45^+^/CD45^-^ systems rather than as an optimized immune-maintenance protocol. Because PBMC subset proportions (including NK cells) can be influenced by culture duration and cytokine supplementation, users may adjust culture conditions for longer study (e.g., IL-2/IL-15 for NK-focused studies) based on experimental goals.

### 2.2 Instruments, software, and general lab equipment

Spectral flow cytometer: Cytek Aurora (5-laser configuration: UV/405/488/561/640 nm); autofluorescence extraction enabled using AF Explorer in SpectroFlo v3.2.1; laser gains optimized daily using QC beads from the corresponding LOT number.

Acquisition and analysis software:

• SpectroFlo for spectral unmixing using single-stain reference controls,
• FlowJo v 10.8.1(BD Biosciences) for data analysis.

Additional equipment: Refrigerated centrifuge (300-500 × g), vortex mixer, pipettes and tips, 5 mL and 15 mL conical tubes, biosafety cabinet (Class II), and 2-8 °C storage protected from light.

### 2.3 Reagents, buffers, and solutions

- Flow Cytometry Staining Buffer: PBS+ 2% BSA
- LIVE/DEAD Fixable Blue Dead Cell Stain Kit (UV): Reconstitute each vial with 50 ul DMSO; prepare a working solution at 1 ul dye in 1 mL PBS; add 250 ul per sample; incubate 30min at RT in the dark.
- CellBlox Plus Blocking Buffer: Add 5 ul per 100 ul final staining volume to reduce non-specific background (including Fc receptor–associated binding that can affect CD16 resolution in PBMCs).
- Super Bright Complete Staining Buffer: Use for antibody master mix and throughout surface staining to prevent polymer-polymer interactions/artifacts.
- FOXP3/Transcription Factor Staining Buffer Set: Prepare Fix/Perm working solution 1:3 (concentrator: diluent); incubate 30–35 min at 2-8°C; wash twice with 1× Permeabilization Buffer.

#### 2.3.1 Antibody mastermix (example, per 1–2 million PBMC)

Prepare the surface master mix in Super Bright Complete Staining Buffer; add this buffer to the tube first. Example optimized volumes per test (sum to ~40–45 ul in buffer): CD25 SB600 2.0 ul; CD69 eFluor450 3.5 ul; TIM-3 SB780 3.5 ul; PD-1 PE 3.5 ul; CD8 PerCP-Cy5.5 2.5 ul; CD4 FITC 1.25 ul; CD45 BUV395 4.0 ul; CD14 SB645 5.0 ul; CD16 NovaFluor Blue 610-70S 2.5 ul; HLA-DR AF700 2.5 ul; CD56 BUV737 2.5 ul; CD11c BUV661 2.0 ul; CLEC4C APC-eF780 2.5 ul; CD83 SB702 5.0 ul, CD141 AF647 2.5 ul. Bring to a final volume of 100 ul per sample with stain buffer, including 5 ul CellBlox Plus Blocking Buffer (to reduce CD16-associated non-specific background and improve signal resolution).

Fluorochrome assignment (including CD4 FITC and FoxP3 PE-Cy7) was determined through iterative panel-wide optimization guided by antigen density, SI, and SSM metrics, and spreading behavior under final workflow conditions, rather than brightness ranking alone. Alternative fluorochromes may be substituted during local adaptation, provided that SI/SSM and spreading characteristics are re-verified.

#### 2.3.2 Controls and reference materials

To ensure reproducibility and spectral accuracy, the following controls are included:

- Single-stain reference controls for each fluorochrome (required for spectral unmixing).
- Fluorescence Minus One (FMO) controls for key markers to set gating boundaries.
- Unstained PBMCs to assess autofluorescence.
- Internal longitudinal control: a cryopreserved PBMC aliquot from a single donor is included in each experiment to assess staining consistency and instrument performance across runs.

## 3 Methods

The objective of this method was to develop a single-tube, 17-color spectral flow cytometry assay capable of simultaneously resolving major PBMC lineages while reporting immune activation (CD25, CD69, CD83), regulatory (FoxP3), and checkpoint (PD-1, TIM-3) programs. The protocol was optimized and validated using freshly isolated PBMCs. Longitudinal reproducibility was monitored using a cryopreserved, single-donor PBMC internal control included in each run with minimal background artifacts and preservation of resolution for dim or rare populations.

Validation focused on:

- Antibody titration for signal-to-noise optimization;
- Suppression of artifacts due to polymer-polymer interactions (using Super Bright buffer) and reduction of CD16-associated non-specific background (using CellBlox Plus Blocking Buffer);
- Spectral unmixing quality with single-stain references and autofluorescence extraction;
- Longitudinal reproducibility using the same-donor PBMC internal control across runs.

### 3.1 Immune cell populations and defining markers

The immune cell subpopulations and their defining markers are summarized in **Table 1**.

**Table 1:** Immune cell populations and defining markers.

Population	Defining markers
Viable leukocytes	CD45^+^, LIVE/DEAD Blue^-^
T cells	CD3^+^
CD4^+^ T cells	CD3^+^ CD4^+^ CD8^-^
CD8^+^ T cells	CD3^+^ CD8^+^ CD4^-^
Tregs	CD3^+^ CD4^+^ CD25^+^ FoxP3^+^
Activated T cells	CD25^+^, CD69^+^ (within CD4^+^ or CD8^+^)
Exhausted T cells	PD-1^+^, TIM-3^+^ (within CD4^+^ or CD8^+^)
NK cells	CD3^-^ CD56^+^
Activated NK cells	CD25^+^, CD69^+^ (within HLA-DR-CD56+)
Exhausted NK cells	PD-1^+^, TIM-3^+^ (within HLA-DR-CD56+)
NKT cells	CD3^+^ CD56^+^
Activated NKT cells	CD25^+^, CD69^+^
Exhausted NKT cells	PD-1^+^, TIM-3^+^
Monocytes (total)	CD14^+^ or CD16^+^
Classical monocytes	CD14^+^ CD16^-^
Intermediate monocytes	CD14^+^ CD16^+^
Non-classical monocytes	CD14^-^ CD16^+^
Exhausted monocytes	TIM3+ (within CD3-HLA-DR+)
DCs	HLA-DR^+^, Lineage^-^ (CD3^-^ CD14^-^ CD16^-^ CD56^-^)
cDCs	CD11c^+^ CLEC4C^-^
pDCs	CLEC4C^+^ CD11c^-^
Activated DCs	CD83^+^ (within CD3-CD14-)

### 3.2 Exact staining protocol

- To stain only PBMC, start from point 13
  1. Harvest GSC-PBMC organoids
  2. Centrifuge organoids at 300g for 4min.
  3. Discard the supernatant completely.
  4. Add 500 µL of Accutase per 1 million cells
  5. Incubate 5–10 min at 37°C. During incubation, gently triturate 5–10 times using a P1000 pipette every 2–3 min to support dissociation.
  6. Optional (recommended for organoids): pass the suspension through a 40 µm cell strainer to remove residual aggregates.
  7. Add 500 µL PBS.
  8. Centrifuge cells 300g, 4min.
  9. Discard the supernatant.
  10. Add 500 µL PBS.
  11. Centrifuge cells 300g, 4min.
  12. Discard the supernatant.
- Viability staining and surface staining
  13. Prepare the viability dye LIVE/DEAD Fixable Blue Dead Cell Stain Kit for UV excitation (Reconstitute with 50 µL DMSO per vial; can be used for 2 weeks).
  14. Prepare working solution: Add 1 µL viability dye to 1 mL PBS, then add 250 µL per sample.
  15. Incubate samples 30min at RT in the dark.
  16. Add 700 µL stain buffer to the sample.
  17. Centrifuge cells 300g, 4min.
  18. Discard the supernatant.
  19. Resuspend cells in X µL stain buffer with 5 µL CellBlox Blocking Buffer.
  20. 100 µL- final volume per sample
  21. Prepare one mastermix cocktail of antibodies in Super Bright Complete Staining Buffer 5 µL/test. Add Super Bright Complete Staining Buffer first to the Falcon tube, then antibodies:
    2.0 µL CD25 SuperBright600
    3.5 µL CD69 eFluor450
    3.5 µL TIM3 SuperBright780
    3.5 µL PD-1 PE
    2.5 µL CD8 PerCP5.5
    1.25 µL CD4 FITC
    4.0 µL CD45 BUV395
    5.0 µL CD14 SB645
    2.5 µL CD16 NovaFluorBlue 610-70S
    2.5 µL HLA-DR AlexaFluor700
    2.5 µL CD56 BUV737
    2.0 µL CD11c BUV661
    2.5 µL CD141 AlexaFluor 647
    2.5 µL CLEC4C APC-eFluor 780
    5.0 µL CD83 SuperBright 702
    2.5 µL CD3 BUV496
  22. Incubate samples for 30min in the dark at 2-8°C.
  23. Add 1 mL stain buffer to samples.
  24. Centrifuge cells 300g, 4min.
  25. Discard the supernatant.
  26. Add 1 mL stain buffer to samples.
  27. Centrifuge cells 300g, 4min.
  28. Discard the supernatant.
  29. Add 150 µL of FOXP3 Fixation/Permeabilization working solution to each tube, then pulse-vortex.
  30. Incubate for 30–35 min at 2-8°C. Protect from light.
  31. Add 300 µL of 1x Permeabilization Buffer to each tube.
  32. Centrifuge the samples at 500g for 4min at RT.
  33. Discard the supernatant.
  34. Add 500 µL of 1x Permeabilization Buffer.
  35. Centrifuge the samples at 500 g for 4 min at RT.
  36. Discard the supernatant.
  37. Resuspend pellet in residual volume of 1x Permeabilization Buffer. This is typically 50 µL after decanting.
  38. Without washing, add the recommended amount of directly conjugated antibody for the detection of intracellular antigen(s) to cells and incubate for at least 30 minutes at room temperature. Protect from light. Nuclear: FoxP3 Pe-Cy7 (1.0µL)
  39. Add 1 mL of 1x Permeabilization Buffer to each tube
  40. Centrifuge cells 500g, 4min.
  41. Discard the supernatant.
  42. Add 1 mL of 1x Permeabilization Buffer to each tube
  43. Centrifuge cells 500g, 4min.
  44. Discard the supernatant.
  45. Resuspend stained cells in an appropriate volume of Flow Cytometry Staining Buffer, 200 μL.
  46. Analyze samples by flow cytometer. Samples can be measured for the next day. Keep them at 2-8°C (Protect from light).

#### 3.2.1 General incubation conditions

- Surface Staining: Cells are incubated with the antibody cocktail at 2-8 °C in the dark for 30 minutes.
- Fixation and Permeabilization: Following surface staining, samples are treated with Foxp3 Fixation/Permeabilization working solution for 30 minutes at 2-8 °C, protected from light.
- Intracellular Staining: After fixation and washing, intracellular markers such as FoxP3 are incubated for 30 minutes at room temperature in the dark.
- Final Resuspension and Acquisition: Cells are resuspended in Flow Cytometry Staining Buffer before acquisition, with the option to measure samples the following day, provided they are protected from light.

#### 3.2.2 Controls required for each experiment

To ensure reproducibility and accuracy, the following controls are essential:

- Single-stain controls for each fluorochrome (used for spectral unmixing in SpectroFlo 3.2.1).
- Unstained PBMC control to assess autofluorescence in SpectroFlo v3.2.1.
- Same-donor PBMC internal control included in every run to assess inter-run variability and instrument performance (batch-effect control).
- Unstained co-culture control (PBMC–CD45^-^ mixed sample) when applying the workflow to mixed systems (especially when comparing PBMC-only vs co-culture samples), to verify context-specific autofluorescence profiles and confirm that autofluorescence extraction does not distort negative populations within key parent gates.

#### 3.2.3 Antibody titration

Antibody titrations were performed for all markers to determine optimal working concentrations for the 17-color spectral panel. Two-fold serial dilutions were tested on PBMCs processed using the final staining workflow (including viability staining) under the same buffering conditions used for the multicolor panel. Final working concentrations were selected as the lowest concentration that produced a near-saturating signal and robust separation of positive and negative populations, while minimizing background staining and preserving resolution of the primary lineage gates; for dim or continuous markers, gate placement was confirmed using matched fluorescence-minus-one controls. For antibodies supplied with a recommended volume per test (e.g., 5 ul/test), dilutions spanning 5 ul to 0.3 ul per test were evaluated. For antibodies supplied at concentration units (µg/mL), dilutions corresponding to 0.5 µg to 30 ng per test were assessed. Final titration outcomes are reported as the selected working volume per test (ul/test).To minimize spreading error, fluorochrome assignment was matched to antigen density: high-expression targets were paired with dim fluorochromes, whereas low-expression or functionally critical markers were assigned to bright, spectrally favorable channels.

### 3.3 Instrument configuration

The instrument configuration used for the 17-color spectral panel is summarized in **Table 2**.

**Table 2:** Instrument configuration.

Fluorochrome	Excitation laser	Peak channel
Brilliant ultraviolet 395	UV	UV2
Brilliant ultraviolet 496	UV	UV7
Brilliant ultraviolet 661	UV	UV11
Brilliant ultraviolet 737	UV	UV14
eFluor450	Violet	V3
SuperBright 600	Violet	V10
SuperBright 645	Violet	V11
SuperBright 702	Violet	V13
SuperBright 780	Violet	V15
FITC	Blue	B2
NovaFluorBlue 610-70S	Blue	B6
PerCP-Cyanine5.5	Blue	B9
PE	Yellow-Green	YG1
PE-Cyanine7	Yellow-Green	YG9
Alexa Fluor 700	Red	R4
APC-eFluor 780	Red	R7

### 3.4 Application and effectiveness

This single-tube, 17-color spectral panel is designed for comprehensive post-stimulation and immune-monitoring readouts across PBMCs. The panel is also directly compatible with mixed co-cultures that include CD45^-^ cells. Using the CD45 backbone, leukocytes (CD45^+^) are cleanly separated from the CD45^-^ compartment, enabling simultaneous immune phenotyping and monitoring of tumor/stromal cells within the same tube. A matched control anchors the CD45^-^ gate and FSC/SSC morphology, ensuring unambiguous separation from leukocytes. In stimulated PBMCs, the assay resolves early and late activation states in CD4^+^ and CD8^+^ T cells, as well as NK and NKT compartments, using CD69 and CD25 as readouts. Simultaneously, it captures checkpoint engagement via PD-1 and TIM-3 expression across both lymphoid and myeloid populations. The panel also identifies regulatory T cells (FoxP3^+^CD25^+^). It distinguishes monocyte subsets (classical, intermediate, and non-classical) and DC populations (classical: cDCs; plasmacytoid: pDCs), with CD83 serving as an activation marker in DCs.

These functional layers are combined into a single staining tube, providing a multidimensional snapshot that would typically need multiple parallel assays. This setup enables efficient immune profiling in both translational and clinical settings, especially when sample volume is limited or higher throughput is required.

Effectiveness was established through a combination of design-in controls and benchmarking. The use of FMO controls allowed for precise gate placement on dim and functionally informative markers, while single-stain references enabled high-quality spectral unmixing. An unstained PBMC sample enabled autofluorescence extraction. Known artifacts, such as CD16-associated non-specific background staining (often Fc receptor–mediated) and polymer–polymer interactions from Super Bright dyes, were actively mitigated using CellBlox Plus Blocking Buffer and Super Bright Complete Staining Buffer, respectively.

### 3.5 Precision, accuracy, and limits of detection/quantification

#### 3.5.1 Repeatability and longitudinal precision

To assess repeatability, an internal PBMC control from the same donor was included in every experimental run. This allowed for real-time monitoring of day-to-day staining consistency and instrument performance. Instrument settings were optimized and recorded for each antibody lot, providing a technical anchor for longitudinal reproducibility without requiring repeated external validation. Marker expression patterns were validated using canonical gating definitions for each lineage and functional subset, ensuring biological relevance and interpretability.

#### 3.5.2 Analytical precision for dim or functional markers

All antibodies were titrated using two-fold serial dilutions on PBMCs. Final working concentrations were selected based on the lowest saturating signal that achieved maximal stain index and FMO-defined gate clarity. To minimize spectral spillover and spreading error, fluorochrome assignment was carefully matched to antigen density. High-expression targets (e.g., CD45) were paired with dim fluorochromes (e.g., BUV395), while low-expression or functionally critical markers (e.g., PD-1, TIM-3, FoxP3) were assigned to bright, spectrally clean channels.

#### 3.5.3 Limits of detection and quantification

As this method is intended for qualitative and semi-quantitative immunophenotyping, strict LoD/LoQ parameters are not defined. Instead, marker positivity is operationally determined using FMO-anchored boundaries, and population-level expression is typically reported using median fluorescence intensity (MFI), frequency, or interquartile range within a standardized gating framework. Autofluorescence subtraction and single-stain spectral references ensure that the positive signal is distinguishable from the background. Importantly, markers such as PD-1 and TIM-3 consistently demonstrated discrete positive populations under standardized gating, demonstrated discrete positive populations under standardized gating in freshly isolated PBMCs; longitudinal stability was monitored in the same-donor internal control.

#### 3.5.4 Limitations and troubleshooting anchors

The primary sources of potential imprecision in spectral flow cytometry, namely CD16-associated non-specific background and polymer-induced spreading from Super Bright dyes, were directly addressed in the protocol. CellBlox Plus Blocking Buffer was included in every staining reaction to reduce CD16-associated non-specific binding and improve resolution within monocyte and NK compartments.

PBMC cryopreservation and aliquoting are commonly used to enable centralized, batched immune monitoring and reduce inter-assay and inter-laboratory variability, as freeze–thaw handling can alter recovery and the apparent detection of selected epitopes in a handling-dependent manner (15, 16). Thus, while several studies using optimized cryopreservation and recovery workflows report only minimal changes in the composition of major PBMC subsets and overall transcriptomic profiles, cryobanking is recommended for centralized, batched analyses (17, 18). Accordingly, consistent thawing/handling, strict viability exclusion, and FMO-anchored thresholds were strictly observed, and a same-donor cryopreserved PBMC aliquot was included as a longitudinal reference control to monitor staining and unmixing performance across runs; this note is provided for troubleshooting/QC rather than to support fresh-versus-cryopreserved biological comparisons (19, 20).

At the same time, Super Bright buffer was used throughout surface staining to prevent fluorescence aggregation and over-spreading. These reagents are essential to the fidelity of dim marker resolution and should be retained in all future applications of the panel. Dim or partially expressed markers remain sensitive to small shifts in antibody titration and spectral overlap, underscoring the importance of consistent instrument settings, proper unmixing, and FMO-defined gates.

## 4 Results

### 4.1 Panel development strategy

The development of this 17-color spectral flow cytometry panel for human PBMCs followed a systematic, iterative optimization process to ensure robust performance, minimal spectral interference, and high biological relevance across diverse immune populations.

The panel was designed to integrate four complementary axes of immune profiling within a single-tube workflow. First, lineage identification is achieved using backbone markers enabling robust separation of major PBMC compartments (e.g., T cells, NK/NKT cells, monocytes, and dendritic cells). Second, activation dynamics are captured using CD69 and CD25, which together enable discrimination of early and late activation states under short stimulation or co-culture conditions. Third, the regulatory axis is represented by FoxP3, which enables the identification of regulatory T cells within the CD4 compartment; including this marker provides an integrated readout of immune activation versus regulation within the same assay. Fourth, checkpoint-associated markers PD-1 and TIM-3 provide a compact view of functional modulation across lymphoid and selected myeloid populations.

CD16 was included to support monocyte subset stratification (classical, intermediate, non-classical) and to capture NK maturation-related states. Plasmacytoid dendritic cells are identified using CLEC4C, selected for specific pDC delineation within the lineage-negative, HLA-DR-positive compartment. Although dendritic cells represent a low-frequency PBMC population, their inclusion supports broad immune-monitoring applications, particularly where antigen-presenting compartments and activation markers (e.g., CD83) are relevant to study design.

Markers were selected to preserve a compact multi-lineage backbone while integrating activation, regulatory, and checkpoint-associated readouts within a single-tube workflow, as detailed in the rationale above.

**Figure 1:**
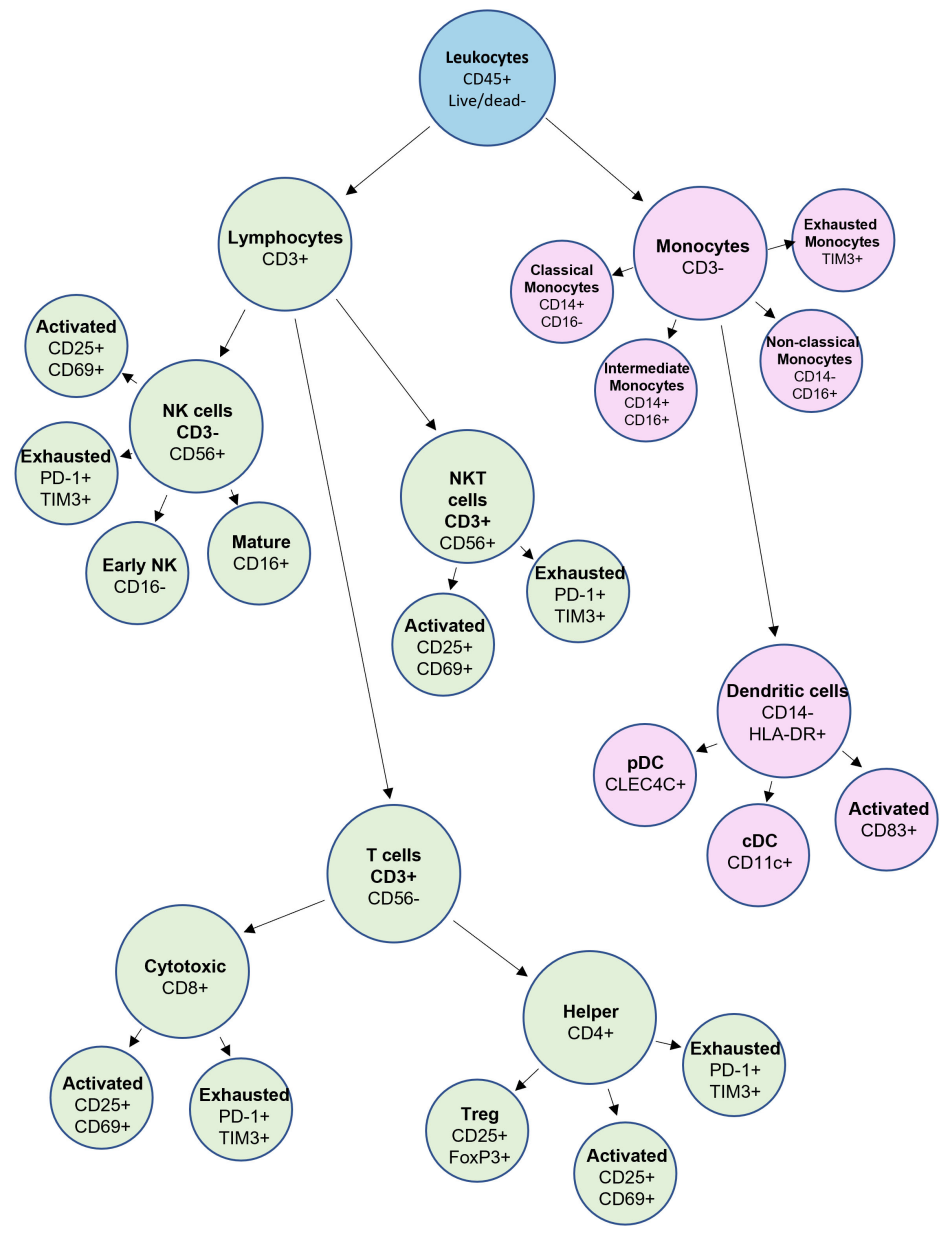
Schematic overview of marker expression on immune cell subsets. Flowchart depicting the hierarchical organization of leukocyte populations identified using the 17-color spectral panel. Starting from live CD45^+^ events, major immune lineages, including T cells, NK cells, NKT cells, monocytes, and DCs, are resolved using backbone markers (e.g., CD3, CD4, CD8, CD14, CD56, HLA-DR). Functional markers are overlaid to define activated (CD25^+^, CD69^+^), exhausted (PD-1^+^, TIM-3^+^), and regulatory (FoxP3^+^) subpopulations. The schematic highlights the panel’s coverage of both adaptive and innate immune compartments and its capacity to provide integrated phenotypic and functional readouts in a single-tube workflow.

To visualize the conceptual layout of the panel, **Figure 1** provides a schematic overview of immune cell subsets resolved by this assay. Starting from live CD45^+^ leukocytes, the gating hierarchy distinguishes major compartments including T cells, NK cells, NKT cells, monocytes, and DCs. Within these lineages, the panel captures functional polarization by integrating activation markers (CD25, CD69), checkpoint-associated markers (PD-1, TIM-3), and the Tregs transcription factor FoxP3. The structure emphasizes the single-tube consolidation of lineage identification and functional profiling across adaptive and innate arms of the immune system.

### 4.2 Characterization of immune cell subsets in peripheral blood mononuclear cells

**Figure 2:**
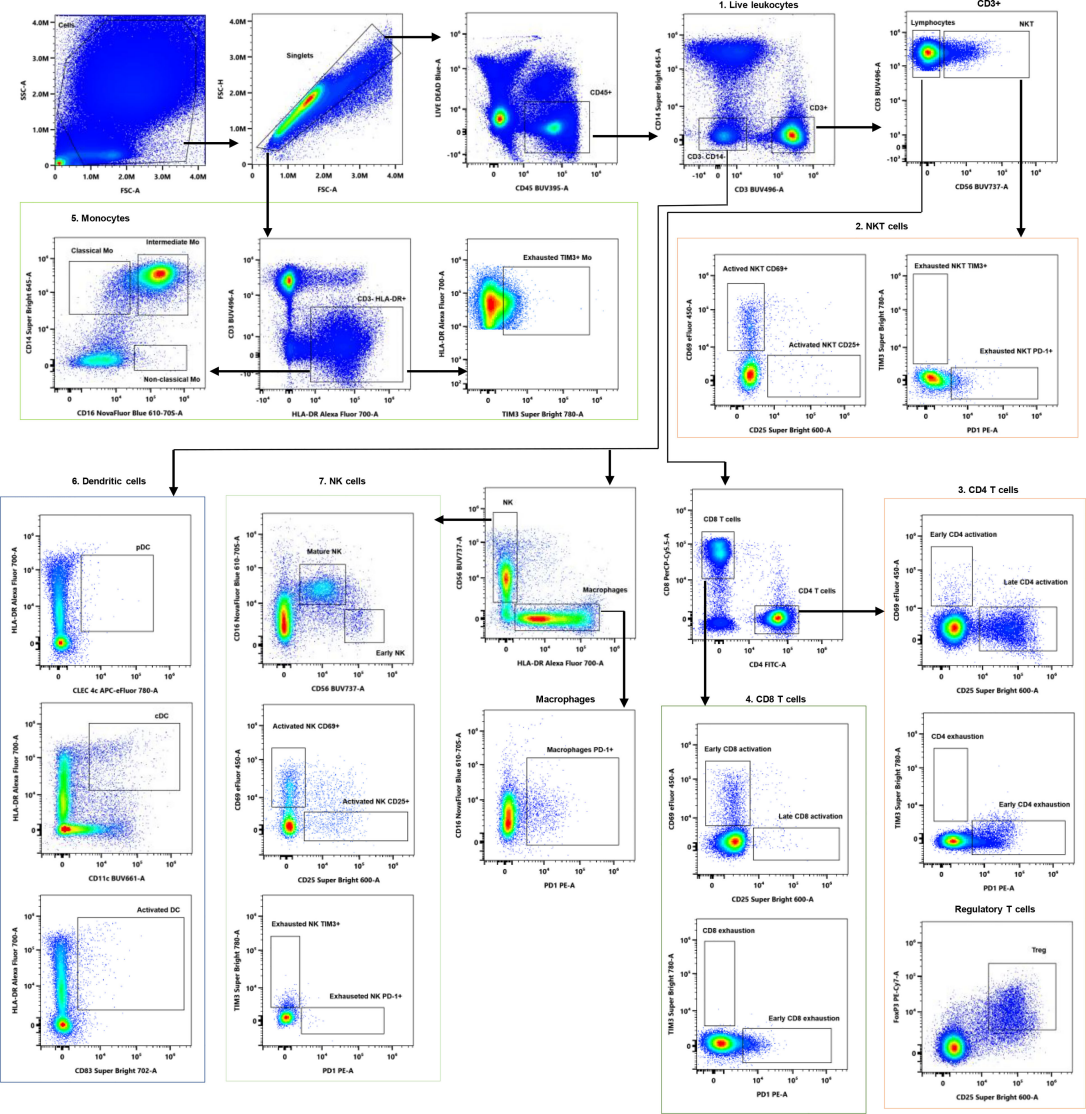
Gating strategy for comprehensive phenotyping of human PBMCs using a 17-color spectral flow cytometry panel.

Manual gating (**Figure 2)** was performed beginning with the exclusion of debris and doublets using SSC-A vs. Time and FSC-H vs. FSC-A plots. Arrows indicate the subsequent gating strategy. Live leukocytes (1) were identified by exclusion of dead cells using LIVE/DEAD Blue dye and by selecting CD45^+^ events. NKT cells (2) were defined as CD45^+^CD3^+^CD56^+^ and were further subdivided into activated NKT cells (CD25^+^ and CD69^+^) and exhausted NKT cells (TIM3^+^ and PD-1^+^). T cells were identified as CD45^+^CD3^+^CD56^-^, and further gated into CD4^+^ (3) and CD8^+^ (4) subsets. Tregs were defined within the CD4^+^ population as FoxP3^+^CD25^+^ cells. CD4^+^ and CD8^+^ T cells were characterized by activation (early: CD69^+^; late: CD25^+^) and checkpoint-associated markers (early exhausted: PD-1^+^; exhausted: TIM3^+^). Monocytes (5) were gated from the CD3^-^HLA-DR^+^ population and classified according to CD14 and CD16 expression into classical (CD14^+^CD16^-^), intermediate (CD14^+^CD16^+^), and non-classical (CD14^-^/dimCD16^+^) subsets. Exhausted monocytes were defined as CD3^-^HLA-DR^+^TIM3^+^. DCs (6) were identified from the CD3^-^CD14^-^ population and further characterized as pDCs (HLA-DR^+^CLEC4C^+^), cDCs (HLA-DR^+^CD11c^+^), or activated (HLA-DR^+^CD83^+^). NK cells (7) were gated from the CD3^-^CD14^-^ population and defined as HLA-DR^-^CD56^+^. The cells were further categorized into mature NK cells (CD56dimCD16^+^) and early NK cells (CD56^+^CD16^-^). NK cells were also assessed for activation (CD69^+^ or CD25^+^) and checkpoint-associated markers (PD-1^+^ or TIM3^+^).

#### 4.2.1 Separation of PBMC from GSC during co-culture staining with a 17-color panel

**Figure 3:**
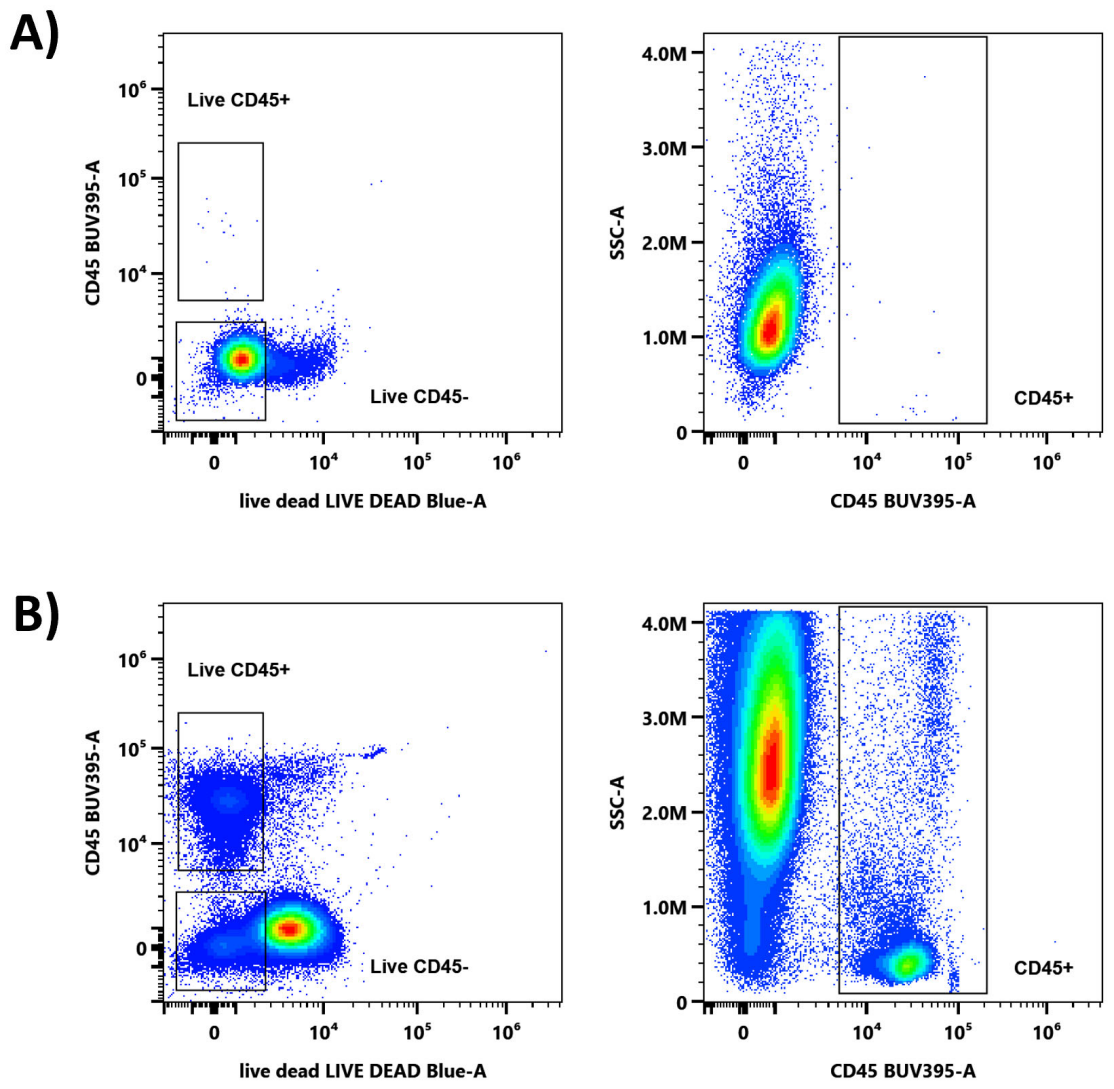
Discrimination of PBMC from GSC using multicolor staining. Plots on the left display staining with CD45 BUV395 together with Live/Dead Blue, while plots on the right show CD45 BUV395 staining alone. **(A) **Staining of the GSC line only with the 17-color panel. **(B)** Staining of the PBMC: GSC co-culture.

To assess whether the multicolor panel could be applied to PBMCs derived from organoids without staining GSCs, the panel was tested on a GSC line alone. As shown in **Figure 3**, none of the antibodies included in the panel stained GSC. Consequently, gating on CD45^+^ cells effectively excluded GSC from the analysis, enabling the specific evaluation of PBMC populations despite their presence in the organoids.

### 4.3 Spectral panel design and fluorochrome assignment

**Figure 4:**
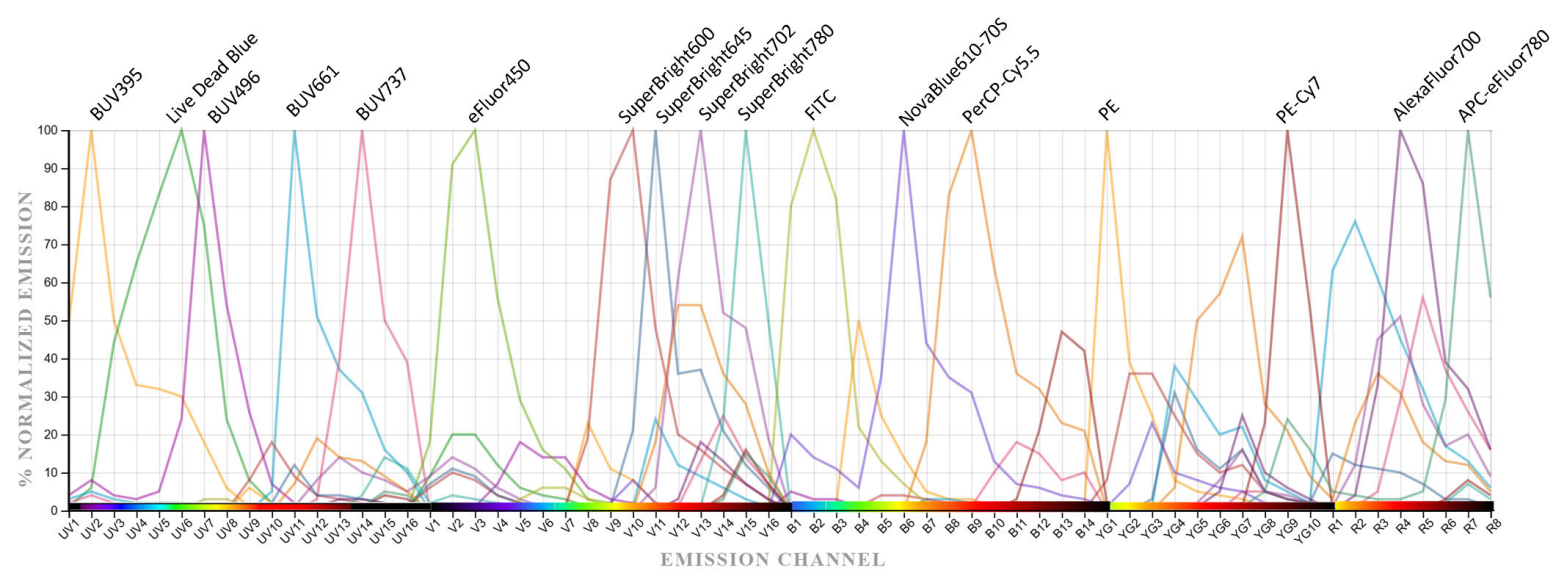
Normalized emission spectra of the 17-color panel on a five-laser spectral cytometer. Overlay of normalized reference spectra for all fluorochromes used in the panel, illustrating spectral distribution across the instrument’s laser configuration and supporting accurate spectral unmixing.

Fluorochrome assignment for the 17-color spectral panel was guided by a combination of antigen density, spectral emission profiles, and compatibility with the five-laser configuration of the Cytek Aurora (UV 355/405/488/561/640 nm). Markers with high expression levels, such as CD45 and CD14, were deliberately paired with dimmer fluorochromes (e.g., BUV395, SB645). In contrast, low-expression or functionally important markers, including PD-1, TIM-3, and FoxP3, were mapped to bright, spectrally clean channels (e.g., PE, SB780, PE-Cy7) to preserve sensitivity and resolution. The panel’s design strategy is illustrated in **Figure 4**, which displays normalized emission spectra for all fluorochromes acquired from full-panel-stained PBMCs. Each dye exhibits a distinct spectral signature, and minimal spectral overlap is observed across the panel, providing the foundation for accurate unmixing and minimal spillover.

The emission profiles reveal key characteristics that informed fluorochrome placement. UV-excited fluorochromes such as BUV395 (CD45), BUV496 (CD3), BUV661 (CD11c), and BUV737 (CD56) displayed narrow, non-overlapping peaks, making them well suited for highly expressed lineage markers. The 405 nm violet laser channel includes eFluor450 (CD69) and multiple Super Bright dyes: SB600 (CD25), SB645 (CD14), SB702 (CD83), and SB780 (TIM-3). As expected, these polymer-based fluorochromes exhibited broader emission profiles, necessitating careful panel balancing to avoid co-expression of markers with overlapping spectra. Dyes excited by the 488 nm blue laser FITC (CD4), NovaFluor Blue 610-70S (CD16), and PerCP-Cy5.5 (CD8) showed relatively distinct peaks with manageable spread. Dyes excited at 561 and 640 nm: PE (PD-1), PE-Cy7 (FoxP3), AF700 (HLA-DR), and APC-eFluor 780 (CLEC4C) displayed broader shoulders, particularly in PE-Cy7 and APC-eFluor 780, which were taken into account during fluorochrome-marker pairing.

These assignments are summarized in **Table 3**, which maps each antibody specificity to its corresponding fluorochrome. The table highlights the balance between core lineage markers (e.g., CD3, CD4, CD8, CD14, CD16, CD56, CD11c) and functional readouts (e.g., CD25, CD69, CD83, TIM-3, PD-1, FoxP3), underscoring the comprehensive scope of the panel. Notably, the emission spectra confirmed that these fluorochromes collectively span the available laser space with minimal overlap, allowing for effective spectral unmixing and minimizing spreading error between co-expressed markers.

**Table 3:** Reagent information.

Specificity	Alternative name	Fluorochrome	Ab clone	Vendor and catalog number	Purpose	Titration
Viability	–	Live Dead UV Blue		Invitrogen L34962	Exclusion of dead cells	1:1000
CD45	Protein tyrosine phosphatase receptor type C	BUV395	HI30	Invitrogen 363-0459-42	Main PBMC marker	4:100
CD3	–	BUV496	UCHT1	BD Horizon™ 612940	T cells	2.5:100
CD8	–	PerCP-Cy5.5	RPA-T8	Invitrogen 45-0088-42	Cytotoxic T cells	2.5:100
CD4	–	FITC	SK3	Invitrogen 11-0047-42	T helper cells	1.25:100
FoxP3	Forkhead box P3	PE-Cy7	PCH101	Invitrogen 25-4776-42	Tregs	1:100
CD56	NCAM	BUV737	TulY56	Invitrogen 367-0566-42	NK cells, NKT cells	2.5:100
CD14	–	SB645	61D3	Invitrogen 64-0149-42	Monocytes	5:100
CD16	FcγRIII	NovaFluor Blue 610-70S	eBioCB16	Invitrogen H025T03B06	Monocytes	2.5:100
CD11c	ITGAX	BUV661	B-ly6	BD Horizon™ 612967	DCs	2:100
HLA-DR	MHC class II	AF700	LN3	Invitrogen 56-9956-42	DCs	2.5:100
CLEC4C	CD303a	APC-eFluor 780	201A	Invitrogen; 47-9818-42	DCs	2.5:100
CD25	IL-2 receptorα chain	SB600	BC96	Invitrogen 63-0259-42	Tregs/activation marker	2:100
CD69	–	eFluor450	FN50	Invitrogen 48-0699-42	Activation marker	3.5:100
CD83	–	SB702	HB15e	Invitrogen; 67-0839-42	Activation marker	5:100
PD-1	CD279	PE	MIH4	Invitrogen 12-9969-42	Checkpoint-associated markers	3.5:100
TIM3	CD366	SB780	F38-2E2	Invitrogen 78-3109-42	Checkpoint-associated markers	3.5:100

Taken together, the combination of emission data and antigen-fluorochrome pairing demonstrates a carefully optimized spectral configuration that enables simultaneous resolution of lineage, activation, regulatory, and checkpoint-associated programs within a single tube. This spectral architecture forms the foundation for all downstream analyses in the panel and supports high-dimensional phenotyping in both research and translational settings.

### 4.4 Spectral similarity and complexity optimization

**Figure 5:**
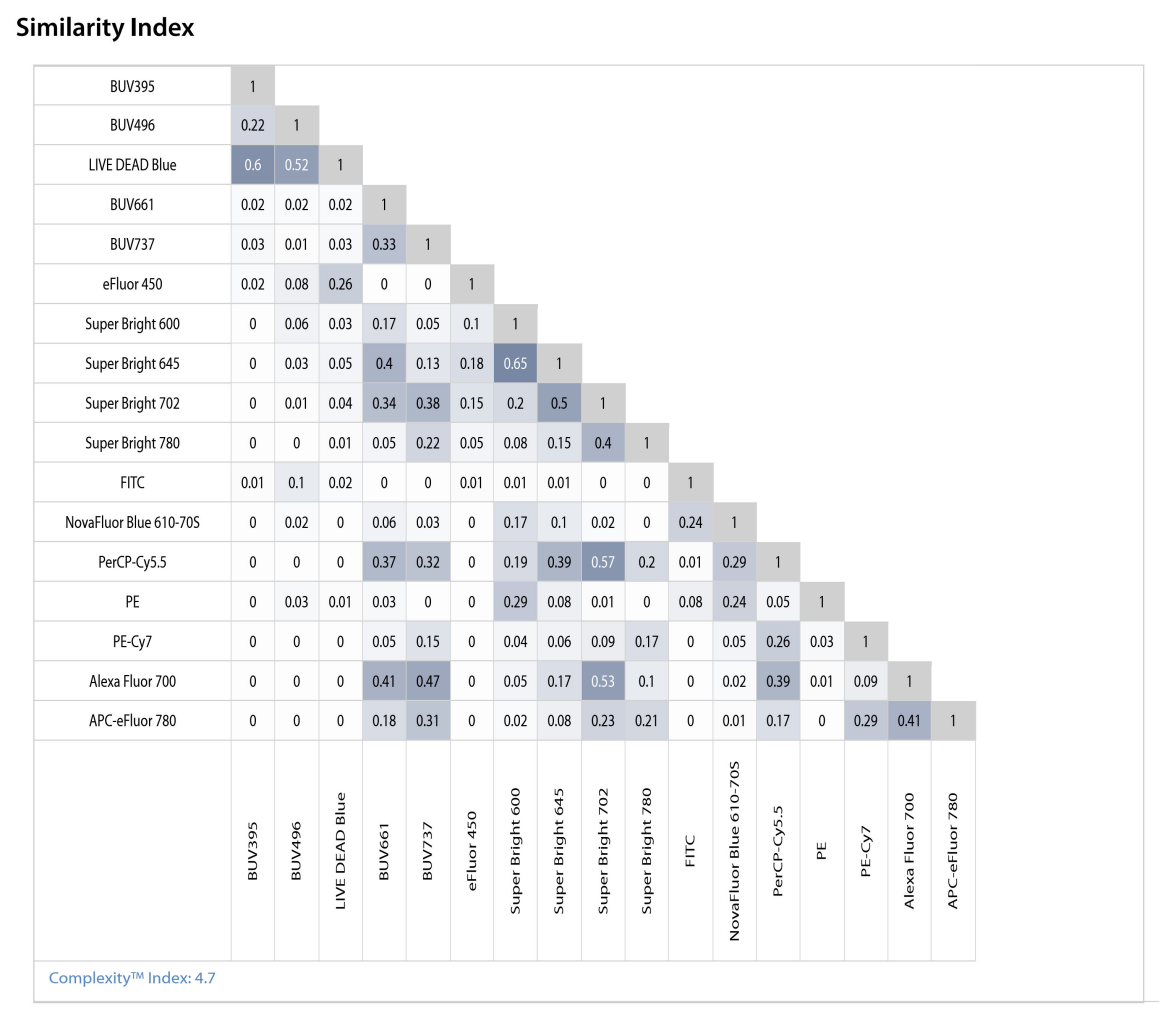
Spectral SI for the 17-color panel. The SI, developed by Cytek and implemented in the SpectroFlo software, ranges from 0 to 1, with 0 indicating no shared spectral characteristics between two fluorochromes and 1 indicating complete spectral identity. Triangular heatmap showing the pairwise similarity (scale 0-1) of reference spectra for all fluorochromes used in the panel. The Complexity Index, calculated in SpectroFlo, reflects interference within a fluorochrome combination; lower values indicate reduced spread and improved resolution. The overall complexity index = 4.7.

To further guide fluorochrome assignment and minimize spectral crosstalk, we generated a similarity index (SI) developed by Cytek and implemented in the SpectroFlo software. The resulting similarity matrix (**Figure 5**) provides a quantitative comparison of spectral overlap across all fluorochromes used in the panel. As expected, members of the Super Bright family (e.g., SB600, SB645, SB702, SB780) exhibit elevated mutual similarity due to their shared polymer backbone, requiring careful separation of co-expressed markers. Moderate similarity was also observed among several red/far-red dyes (e.g., PE-Cy7, AF700, APC-eFluor 780), while UV-excited dyes such as BUV395 and BUV496 displayed less similar emission profiles. These insights enabled rational assignment of dim or functionally critical targets (e.g., PD-1, TIM-3, FoxP3) to low-similarity channels, preserving resolution. The final panel configuration achieved a Complexity Index of 4.7, calculated in SpectroFlo, consistent with manageable spectral overlap after optimization and unmixing. The SI and the complexity index together provide a measure of the overall extent of interferences caused by spillover between and among fluorochromes.

### 4.5 Fluorochrome spread and channel resolution

To evaluate how each fluorochrome affected resolution across the panel, we used an SSM calculated for all fluorochromes. The matrix quantifies how much each fluorochrome (row) increases signal width in other detection channels (columns) after spectral unmixing. This readout is critical for identifying combinations likely to impair separation between positive and negative populations, especially when co-expressed.

**Figure 6:**
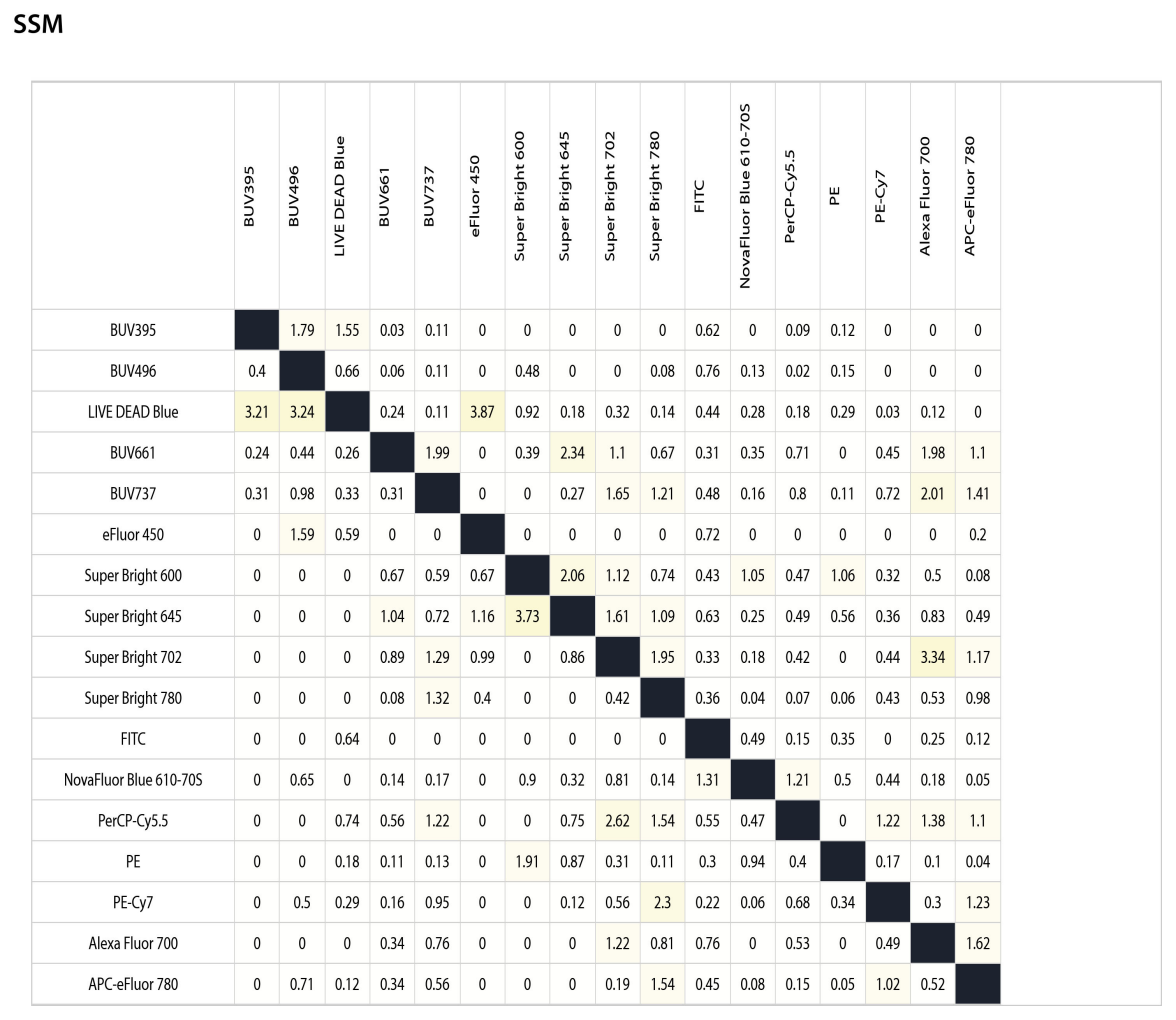
SSM for the 17-color spectral panel. Matrix values quantify the extent to which the fluorochrome in each row increases the spread in each detector channel (column) following spectral unmixing of single-stained controls. Owner values indicate reduced spread and improved resolution. The SSM guided fluorophore-antigen pairing by identifying fluorochromes with low spread into key functional channels (e.g., PD-1, TIM-3, FoxP3) and by avoiding co-expression of markers on fluorochromes with high mutual spread (e.g., Super Bright 645 and PE-Cy7).

As shown in **Figure 6**, the most pronounced spreading was observed for polymer-based dyes, notably LIVE/DEAD Blue (e.g., 3.87 spread into eFluor450), Super Bright 645 (3.73 into Super Bright 600), Super Bright 702 (3.34 into Alexa Fluor 700), PerCP-Cy5.5 (2.62 into Super Bright 702) and BUV661 (2.34 into Super Bright 645). Tandem dyes such as APC-eFluor 780, Alexa Fluor 700, and PerCP-Cy5.5 also exhibited moderate cross-channel spread, consistent with their broader, less stable emission profiles.

These data directly informed decisions on fluorophore-antigen pairing. Markers expected to have low expression (e.g., PD-1, TIM-3, FoxP3) were deliberately assigned to low-spread channels, such as PE, SB780, and PE-Cy7, but only when those channels showed minimal incoming spread from co-expressed markers. For example, CD83 (Super Bright 702) was positioned away from PE-Cy7 and APC-eFluor 780 due to potential spillover. The matrix also reinforced that UV-excited dyes, such as BUV395 and BUV496, exhibit minimal spectral overlap, supporting their use as abundant markers, such as CD45 and CD3.

Overall, the Spread Spillover Matrix (SSM) provided critical quantitative insight that complemented the spectral similarity matrix, allowing us to minimize unintended channel interference and preserve resolution for dim and functionally relevant markers.

### 4.6 Antibody titration and signal optimization

**Figure 7:**
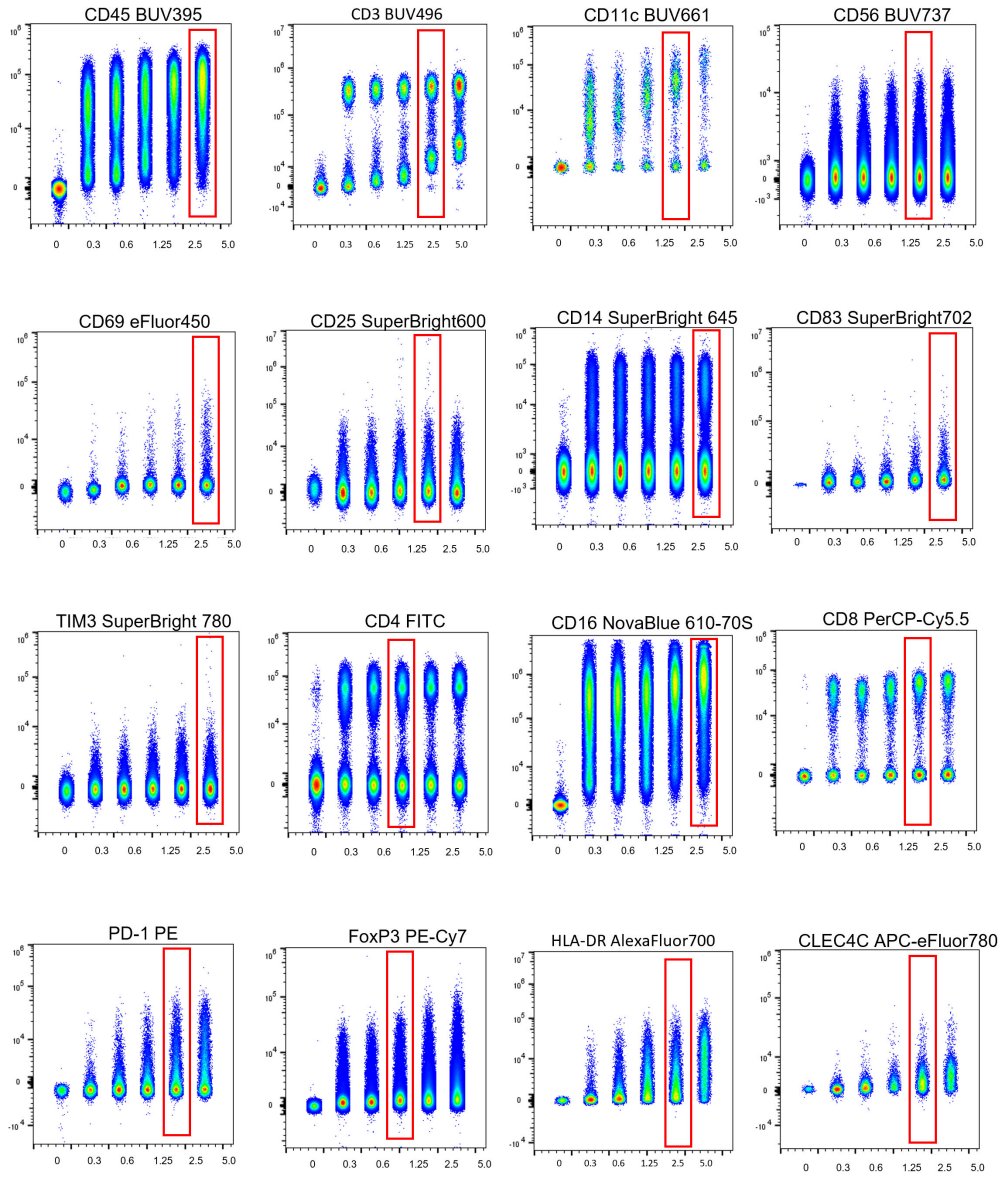
Antibody titration curves for all panel markers. Two-fold serial antibody titrations were performed to determine optimal staining conditions for each marker. Final working concentrations were selected to maximize the separation of positive and negative populations while minimizing background.

To determine optimal antibody concentrations for all 17 markers, we performed serial two-fold titrations on PBMCs. **Figure 7** displays representative titration curves across the full panel, where increasing antibody concentration is plotted against staining intensity. For pre-diluted reagents (e.g., 5 ul/test), dilutions ranged from 5.0 to 0.3 ul/test. All titrations were analyzed in concatenated files using FlowJo v10.8.1 to facilitate direct comparison of staining index and background across conditions.

The optimal concentration for each antibody was determined as the lowest volume required to achieve signal saturation with minimal background, guided by FMO controls. As shown in **Figure 5**, markers with high antigen abundance, such as CD45-BUV395 and CD14-SB645, required relatively high antibody volumes (5 μL/test) to preserve resolution across a wide dynamic range. In contrast, dim or intracellular markers, including FoxP3-PE-Cy7 (1.0 μL), PD-1-PE (3.5 μL), and TIM-3-SB780 (3.5 μL), required careful titration to ensure sufficient signal without excessive background. These targets were assigned to bright, spectrally distinct channels to maximize detection sensitivity, and gate placement was verified using FMO controls. This titration-based strategy ensured consistent staining performance across diverse antigen densities and fluorescence intensities.

This process was vital for markers conjugated to polymers (e.g., Super Bright 645, 702, 780), which can exhibit non-linear background and spreading behavior at suboptimal concentrations. Super Bright Complete Staining Buffer was used to reduce polymer-related interactions in complex multicolor mixtures, and CellBlox Plus Blocking Buffer was included during surface staining to reduce CD16-associated non-specific background (notably for CD16 NovaBlue 610-70S).

Where multiple clones were available (e.g., for PD-1 and TIM-3), reagent selection was based on side-by-side comparison of brightness, specificity, and compatibility with spectral unmixing. The final panel achieved consistent, high-quality staining across all 17 channels, with titrations supporting clear gating, reproducible signals, and minimal spreading.

### 4.7 FMO controls support the gating of dim/functional markers

**Figure 8:**
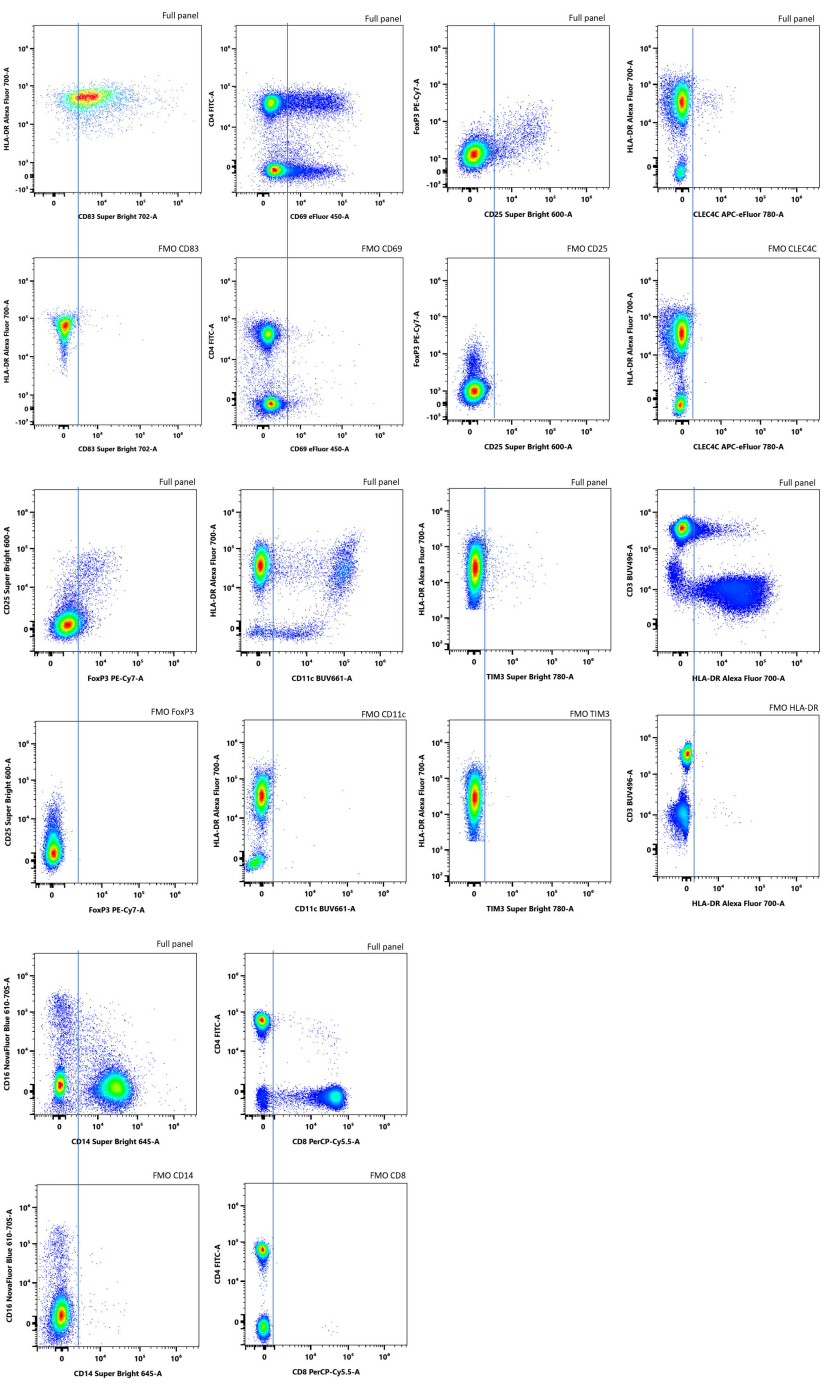
FMO controls anchor gate placement for dim/functional markers. Representative biaxial density plots of PBMCs stained with the full 17-color panel (upper panels) alongside matched FMO plots (down panels) for selected markers. Examples include activation (CD25, CD83), lineage/myeloid (CD14, CD8, CD56), and checkpoint markers.

To rigorously define gating boundaries for dim or functionally critical markers, we used FMO controls for each relevant antibody. **Figure 8** shows representative biaxial plots comparing full 17-color panel staining to the matched FMO condition where the indicated antibody was omitted.

In several channels, particularly CD25-SB600, CD83-SB702, and TIM-3-SB780, the FMO control was critical for accurately distinguishing true positives from background or spread-associated noise. For instance, in the CD83 and TIM-3 plots, inclusion of the antibody caused a subtle but biologically meaningful shift, and without FMO-defined baselines, gating would risk either over- or under-calling expression. The FMOs for CD14 and CD8 showed well-separated negative populations, affirming minimal background and confirming the appropriateness of the assigned fluorochromes.

The FMO-defined boundary helped optimize gate placement and ensured reproducibility across donors. This was especially important for rare populations (e.g., FoxP3^+^ Tregs) or low-expression markers, where misclassification can significantly skew population frequencies or functional interpretations.

This approach was especially critical for dim or functionally variable markers such as PD-1, TIM-3, FoxP3, and CLEC4C, where expression levels were modest, and signal could be distorted by spillover or biological heterogeneity. In channels such as TIM-3-SB780, FoxP3-PE-Cy7, and CLEC4C-APC-eFluor 780, full-panel conditions showed slightly broader distributions and elevated background. Nonetheless, the FMO-based boundaries provided a reproducible anchor point for defining true positives, reducing ambiguity in gate placement.

Other markers, including CD25, CD69, CD11c, and HLA-DR, displayed robust signal separation, and their gates remained stable across conditions. This consistency demonstrates the utility of FMO-defined boundaries for standardizing analysis in high-dimensional panels, particularly when working with longitudinal samples, low-frequency subsets, or multi-operator data pipelines (**Figure 8**).

To maximize analytical precision, antibody titration was performed by serial dilution, and gates were set with thresholds derived from FMO, ensuring objective placement of boundaries on continuity markers. This approach enabled clear separation of weak or functional reads, particularly TIM-3, PD-1, and FoxP3, by combining optimal antibody concentrations with bright fluorochromes, validating resolution using single-dye references and autofluorescence extraction, and by validating resolution using single-dye references. Most importantly, FMO-defined gates were transferable across donors, stimulation conditions, and data-acquisition days, supported by a same-donor PBMC internal control that maintained consistency across series. The workflow is compatible with short-term stimulation assays, supports longitudinal monitoring without gate readjustment, and enables consistent, repeatable measurement of activation, regulation, and checkpoint-associated states in translational studies when standardized quality-control checkpoints and longitudinal internal controls are applied.

By combining FMO anchoring with full-panel application, this gating strategy minimized operator bias, enabled comparability across datasets, and preserved biological accuracy in populations sensitive to small shifts in signal intensity.

Markers such as PD-1, TIM-3, and FoxP3 may display low baseline expression in healthy donor PBMCs and are sensitive to spreading error and small shifts in unmixing or background. Accordingly, reliable interpretation of dim channels requires (i) matched FMO controls for gate placement, (ii) routine verification of single-stain reference controls and autofluorescence extraction, and (iii) monitoring of spread in adjacent channels. For low-frequency populations (e.g., FoxP3^+^ Tregs and pDCs), adequate event acquisition within parent gates is recommended to stabilize estimates of subset frequency and MFI. Together, these measures minimize false positives and false negatives and improve reproducibility across runs.

### 4.8 Effect of the spread resolved with FMO

**Figure 9:**
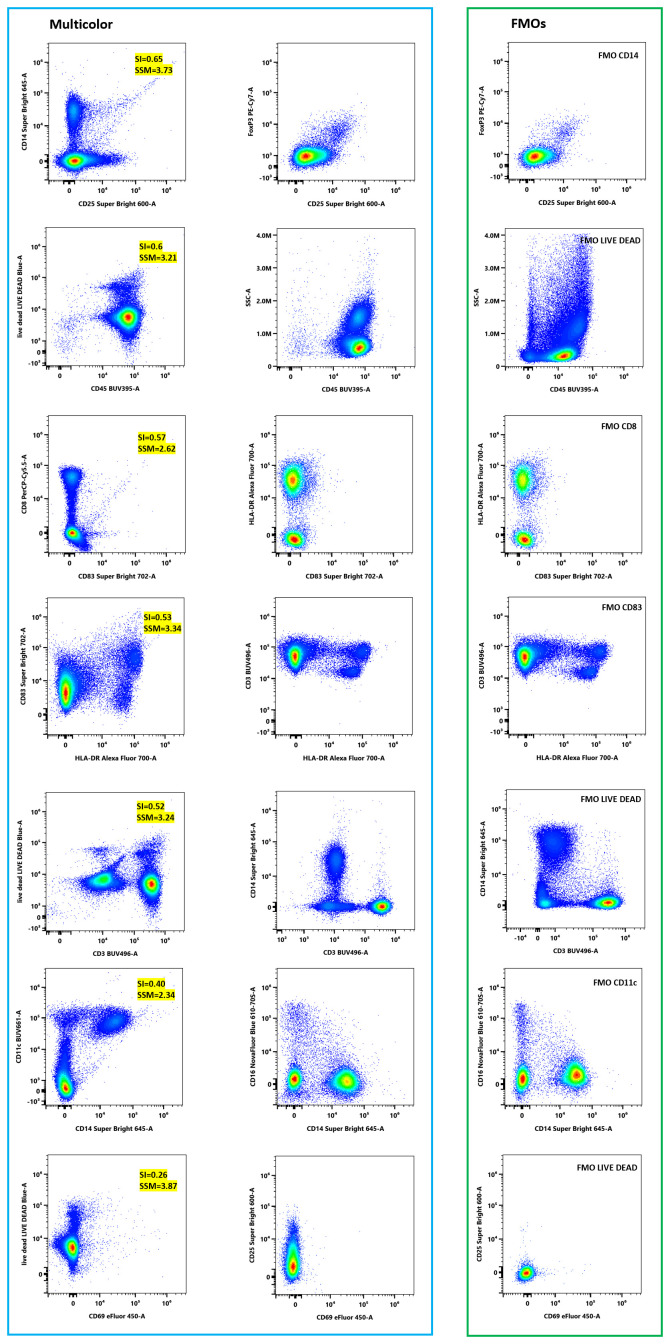
Effect of spread on the resolution of a specific marker. The impact of spread from highly overlapping fluorochromes was assessed by comparing fully stained multicolor samples (blue frame) with matched FMO controls (green frame). Left panels show the fluorochrome pair generating spread (y-axis → x-axis) with SI and SSM values (yellow boxes), while the middle vertical and right vertical panels show the same gated population in the multicolor sample and the corresponding FMO control, respectively.

Based on the SSM, the most pronounced spreading was observed for the following fluorochrome pairs: Live Dead Blue into eFluor 450 (SSM = 3.87), Super Bright 645 into Super Bright 600 (SSM = 3.73), Super Bright 702 into Alexa Fluor 700 (SSM = 3.34), Live Dead Blue into BUV496 (SSM = 3.24), Live Dead Blue into BUV395 (SSM = 3.21), PerCP-Cy5.5 into Super Bright 702 (SSM = 2.62), and BUV661 into Super Bright 645 (SSM = 2.34). To evaluate the effect of this spreading on population resolution, FMO controls for highly overlapping fluorochromes were compared with their corresponding multicolor samples. Fluorochromes were selected for analysis when their SSM value exceeded 2.3. As illustrated in **Figure 9**, population resolution was nearly identical between multicolor samples and their respective FMO controls, indicating that spread did not significantly impair discrimination of the examined subsets. In each panel, the plots in the left column display the fluorochrome pairs, with the fluorochrome on the y-axis introducing spread into the one on the x-axis. The corresponding SI and SSM values are shown in the yellow box. Plots in the middle column illustrate the gated population in the multicolor sample. In contrast, the plots in the right column show the same gated population in the matched FMO control (i.e., all markers included except the x-axis marker). As the x-axis fluorochrome is omitted in the FMO, any events appearing in the x-positive region (e.g., the upper-right quadrant) represent spread, allowing direct assessment of whether spread compromises population discrimination. The blue frame highlights the multicolor samples, whereas the green frame delineates FMO samples.

## 5 Discussion

In this study, we present a robust, single-tube 17-color spectral flow cytometry panel for comprehensive profiling of human PBMCs, integrating lineage, activation, regulatory, and checkpoint markers within a single assay. This approach reduces the need for multi-tube workflows in settings where sample volume is limited, while maintaining interpretable lineage and functional readouts that limit multi-tube strategies. Through iterative optimization of fluorochrome assignment and workflow using FMO controls for poorly visible targets (such as PD-1, TIM-3, FoxP3), single dye references for high-quality separation, autofluorescence modelling and extraction, and mitigation of known artifacts using CellBlox Plus Blocking and Super Bright buffers, we achieved reliable detection of rare and faint signals without compromising the resolution of core cell line gates. The assay was consistently performed on PBMCs, with an internal control harvested from the same donor to ensure comparability across experiments. This also translated into a smooth transition to co-cultures containing CD45^-^ cells without changing the antibody cocktail. In practice, this reduces sample consumption and labor time while enabling consistent, longitudinal immune monitoring suitable for translational processes. Therefore, we consider this panel not only a ready-to-use solution but also a transparent blueprint that adapts with minimal re-optimization. We are confident in its reproducibility, as it is supported by an internal control design intended to enable inter-run monitoring; quantitative inter-run benchmarking is described as a recommended implementation step.

Spectral flow cytometry enables expanded multiplexing capacity; however, increased panel complexity requires rigorous optimization and control design over conventional compensation-based methods, as it records the complete emission profile of each fluorochrome and uses computational dye separation to distinguish overlapping peaks, thereby improving the resolution of faint targets and increasing the possible panel size without excessive scatter. This reduces the classic pitfalls associated with compensation/overfill and allows the inclusion of spectrally similar pairs that would be impractical in traditional systems. We implemented these benefits by (i) using spectral similarity indices and SSM to guide fluorochrome and antigen pairing (prioritizing bright dyes for low-abundance epitopes and minimizing pair spreading in critical channels) and (ii) validating with single dye references to separate and control FMO to place a gate on continuous markers. To address known limitations, Super Bright Complete Staining Buffer was used to minimize polymer-related interactions that can increase background and spreading in complex panels, and CellBlox Plus Blocking Buffer was used to reduce non-specific background signals (particularly those affecting CD16 resolution). These steps, aligned with current spectral cytometry guidance on panel optimization and control design, supported stable marker separation and repeatable performance across runs.

During co-culture of GSCs with PBMCs, early immune activation profoundly affects forward and side-scatter properties due to increased cell size, granularity, and autofluorescence. These changes lead to partial overlap between immune and tumor cell populations in the FSC/SSC space, rendering morphological discrimination unreliable. To preserve the accuracy of immune versus tumor compartment analysis, gating was anchored in CD45 expression, allowing clear separation of CD45^+^ hematopoietic cells from CD45^-^ tumor cells (21).

The use of spectral autofluorescence extraction, combined with careful doublet exclusion and optimized dissociation procedures, further minimized signal spillover and cross-contamination between compartments (13, 22, 23). Consequently, activation and checkpoint-associated markers (CD69, CD25, PD-1, TIM-3, FoxP3) were quantified exclusively within genuine PBMCs, while GSCs remained confined to the lineage-negative CD45^-^ gate (24, 25).

Despite its strengths, this workflow has several technical limitations. First, its performance strongly depends on the quality and stability of spectral unmixing and reference controls. Inaccurate single-stain references or suboptimal autofluorescence extraction can distort negative populations and increase background noise, potentially leading to false-positive events. Routine verification of spectral references and periodic re-titration of antibodies are therefore essential to ensure long-term reproducibility across runs and instruments (19, 26).

Second, some fluorochromes are inherently more prone to artifacts. Polymer-based dyes, such as the Super Bright series, can display polymer–polymer interactions and elevated background when combined in complex multicolor panels. These effects can be mitigated by using manufacturer-recommended blocking buffers and by carefully balancing fluorochrome combinations. Similarly, tandem dyes, including APC-based conjugates (e.g., APC-eFluor 780), are susceptible to light- and reactive oxygen species-induced degradation, which can generate a spurious signal from the donor fluorophore. Strict light protection and antibody lot validation are necessary to maintain signal fidelity (27, 28).

Finally, the approach requires access to a spectral flow cytometer and operator familiarity with unmixing workflows, which may limit its immediate implementation in laboratories using conventional compensation-based systems. However, as spectral cytometry becomes more accessible, these barriers are expected to diminish through standardized training and cross-platform protocols (19). Nevertheless, these limitations can be systematically minimized through standardized reference panels, robust spectral calibration, and training protocols, which are increasingly implemented in multi-site studies.

Spectral flow cytometry is increasingly adopted for immune monitoring because it enables higher-parameter measurements within practical single-tube workflows while maintaining interpretability through rigorous controls (29). In this context, the present Methods resource is positioned as technology- and vendor-agnostic: the key contribution is the standardized design-and-QC framework (including SI/SSM-informed optimization, systematic titration, autofluorescence handling, and FMO-anchored gating) rather than dependence on a single instrument model (12). Compared with conventional filter-based systems, spectral approaches are generally less constrained by fixed filter configurations, which can make panel adaptation across spectral instruments more tractable (29). At the same time, portability is not automatic and requires instrument-specific reference controls and verification of unmixing/spreading performance during implementation (12, 30).

A deliberate design choice was the use of a fixed, single-tube marker set to support cross-condition and longitudinal comparability and to enable exploratory, high-dimensional analyses in settings where the relevant immune signatures may not be known *a priori* (e.g., organoid and immune–tumor co-culture studies) (29, 30). This “always-measured” structure avoids analytical gaps introduced by swapping markers between experiments and facilitates consistent integration of lineage resolution with activation, regulatory, and checkpoint-associated readouts. Where specific readouts are not required in a given application (e.g., FoxP3-based Treg definition), the workflow can be implemented in a streamlined surface-only variant, with the understanding that the corresponding intracellular endpoint will not be captured in that mode.

The proposed 17-color spectral panel provides a versatile platform for immune-monitoring across multiple translational contexts. It enables comprehensive analysis of activation and checkpoint markers following immune stimulation, making it particularly suitable for assessing immune responses in immunotherapy trials and vaccine studies (19, 31, 32). The ability to combine phenotypic and functional readouts within PBMCs facilitates detailed profiling of post-stimulation dynamics, including T cell activation and exhaustion.

The panel was validated for reproducible PBMC immunophenotyping within a standardized quality-control framework, supporting multicenter and longitudinal studies where harmonized workflows and consistent performance are critical (18, 33). In such settings, rigorous pre-analytical standardization, inclusion of longitudinal internal controls, and predefined gating anchors are commonly used to minimize batch effects and enable cross-run comparability. This workflow integrates these elements to support consistent readouts in large-scale studies that require standardized controls and harmonized gating strategies.

Significantly, the single-tube design minimizes sample input requirements while maintaining analytical depth, making it particularly advantageous for samples with limited blood volume (e.g., pediatric) (34, 35). In mixed co-culture systems containing both hematopoietic and non-hematopoietic components, the inclusion of CD45 as a lineage discriminator preserves analytical integrity, enabling simultaneous immune phenotyping and tumor-compartment tracking within a single acquisition. While certain technical constraints remain inherent to spectral cytometry, the optimized design of this panel allows broad applicability across a range of translational and clinical contexts.

Building on this panel’s established performance, future developments can further extend its performance and expand its analytical and clinical scope. The incorporation of functional readouts, such as intracellular cytokine staining (ICS) for IFN-γ, TNF, or IL-2, and proliferation markers like Ki-67, would expand its analytical scope to encompass activation, multifunctionality, and clonal expansion. These additions could be integrated into established ICS harmonization frameworks, facilitating consistent application across laboratories and clinical immuno-monitoring studies (36–39).

Another promising direction is adapting the staining procedure for whole-blood assays, which would streamline sample handling, reduce processing time, and improve clinical feasibility in large, multicenter trials. Whole blood ICS approaches have already demonstrated robust reproducibility and compatibility with spectral cytometry workflows (26).

Advances in computational cytometry also open new avenues for analysis. The integration of machine learning-based pipelines for automated gating and quality control, such as segmentation-based, reference-driven, or anomaly detection frameworks, can standardize gate placement, enhance detection of rare events, and ensure reproducibility across cohorts and time points (40, 41).

Finally, extending these analytical and functional modules to organoid systems containing tumor and immune cells could provide a powerful platform for studying cellular interactions within disease-relevant microenvironments. In this setting, CD45-based partitioning can be complemented by cytokine and proliferation readouts, enabling simultaneous assessment of immune functionality and tumor dynamics in contexts such as cancer, autoimmunity, and infection.

In summary, this work delivers a single-tube, 17-color spectral panel with a practical optimization and control framework that supports reproducible PBMC immuno-monitoring across donors, sample states, and mixed co-cultures containing CD45^-^ cells. By combining a CD45-anchored backbone with activation, regulatory, and immune checkpoint readouts, the assay enables streamlined, sample-sparing profiling that avoids a population-resolution trade-off. The panel is intended as both a deployable reagent set and a transferable blueprint for adapting fluorochrome-antigen pairing and control design as targets or applications evolve. This includes future extensions to functional add-ons such as intracellular cytokine staining and proliferation markers, as well as tumor–immune organoid workflows.

## 6 Nomenclature

### 6.1 Resource identification initiative

Key resources and reagent identifiers used in this study are summarized in **Table 4**.

**Table 4:** Key resources and reagents.

Reagent or resource	Source	Identifier
Antibodies		
BD Horizon™ BUV496 Mouse Anti-Human CD3 clone UCHT1	Invitrogen	#612940, (RRID: AB_2870222)
Anti-human CD8a Monoclonal Antibody PerCP-Cyanine5.5 clone RPA-T8	Invitrogen	#45-0088-42, (RRID: AB_1582255)
CD25 Monoclonal Antibody (BC96), Super Bright™ 600, eBioscience™	Invitrogen	#63-0259-42, (RRID: AB_2637187)
Anti-human CD69 Monoclonal Antibody eFluor450 clone FN50	Invitrogen	#48-0699-42, (RRID: AB_2574025)
CD279 (PD-1) Monoclonal Antibody (MIH4), PE, eBioscience™	Invitrogen	#12-9969-42, (RRID: AB_10736473)
CD366 (TIM3) Monoclonal Antibody (F38-2E2), Super Bright™ 780, eBioscience™	Invitrogen	#78-3109-42, (RRID: AB_2724091)
CD45 Monoclonal Antibody (HI30), Brilliant ultra Violet™ 395, eBioscience™	Invitrogen	#363-0459-42, (RRID: AB_2920949)
CD4 Monoclonal Antibody (SK3 (SK-3)), FITC, eBioscience™	Invitrogen	#11-0047-42, (RRID: AB_1272074)
CD14 Monoclonal Antibody (61D3), Super Bright™ 645, eBioscience™	Invitrogen	#64-0149-42, (RRID: AB_2662458)
Anti-human CD16 Monoclonal Antibody NovaFluorBlue 610-70S clone eBioCB16 (CB16)	Invitrogen	#H025T03B06, (RRID: AB_2910727)
HLA-DR Monoclonal Antibody (LN3), Alexa Fluor™ 700, eBioscience™	Invitrogen	#56-9956-42, (RRID: AB_1907427)
CD56 (NCAM) Monoclonal Antibody (TulY56), Brilliant ultra Violet™ 737, eBioscience™	Invitrogen	#367-0566-42, (RRID: AB_2895975)
BD Horizon™ BUV661 Mouse Anti-Human CD11c Clone B-ly6 (RUO)	BD Horizon	#612967, (RRID: AB_2870241)
FOXP3 Monoclonal Antibody (PCH101), PE-Cyanine7, eBioscience™	Invitrogen	#25-4776-42, (RRID: AB_10804638)
CD303a Monoclonal Antibody (201A), APC-eFluor™ 780, eBioscience™	Invitrogen	#47-9818-42, (RRID: AB_2784717)
CD83 Monoclonal Antibody (HB15e), Super Bright™ 702, eBioscience™	Invitrogen	#67-0839-42, (RRID: AB_2802455)
Biological samples		
Buffy coats obtained from healthy donors	Regional Blood Center, Warsaw	Approval number WAW/111/2021
Chemicals, peptides, and recombinant proteins		
Neurobasal™ Medium	Gibco	21103049
B-27™ Supplement (50X), minus vitamin A	Gibco	12587010
Human Heat Stable bFGF Recombinant Protein 100ug	Gibco	# PHG0369
Human EGF Recombinant Protein 100ug	Gibco	PHG0311
Penicillin-Streptomycin (10,000 U/mL)	Gibco	15140122
GlutaMAX (100x)	Gibco	35050-061
Bambanker™ - freezing medium 120ml	GC LYMPHOTEC INC.	BB01
StemPro Accutase 100ml	Gibco	A11105-01
Lymphoprep™ 500ml	StemCell	7851
CellBlox™ Plus Blocking Buffer	ThermoFisher Scientific	C001T03F01
ultraComp eBeads™ Compensation Beads	Invitrogen	01-2222-41
LIVE/DEAD™ Fixable Blue Dead Cell Stain Kit, for UV excitation	Invitrogen	L34962
RPMI 1640 Medium, GlutaMAX™ Supplement	Gibco	61870010
Experimental models: Cell lines		
Patient-derived glioblastoma stem-like cells (GSCs)	Brigham and Women’s Hospital	(42, 43)
Software and algorithms		
SpectroFlo 3.2.1	Cytek Biosciences	(RRID: SCR_025494)
FlowJo v10	BD Bioscience	https://www.flowjo.com/, (RRID: SCR_008520)
Other		
Corning^®^ Falcon^®^ Round Bottom Test Tubes	Merck	CLS352054
T75 Nunclon Sphera EasYFlask	ThermoFisher Scientific	174952
Nunclon Sphera 12-Well Plate, case of 7	ThermoFisher Scientific	174931
Nunclon™ Sphera™ Dishes 6-Well Plate	ThermoFisher Scientific	174932

## Data Availability

The raw data supporting the conclusions of this article will be made available by the authors, without undue reservation.
